# Rare disease education in Europe and beyond: time to act

**DOI:** 10.1186/s13023-022-02527-y

**Published:** 2022-12-19

**Authors:** Birute Tumiene, Harm Peters, Bela Melegh, Borut Peterlin, Algirdas Utkus, Natalja Fatkulina, György Pfliegler, Holm Graessner, Sanja Hermanns, Maurizio Scarpa, Jean-Yves Blay, Sharon Ashton, Lucy McKay, Gareth Baynam

**Affiliations:** 1 Institute of Biomedical Sciences, Faculty of Medicine, Vilnius University, Vilnius, Lithuania; 2 Dieter Scheffner Center for Medical Education and Educational Research, Dean’s Office of Study Affairs, Charité - Universitätsmedizin, Berlin, Germany; 3 Association of Medical Schools in Europe e.V., Berlin, Germany; 4 Department of Medical Genetics, and Szentagothai Research Center, University of Pecs, School of Medicine, Pecs, Hungary; 5 Clinical Institute of Genomic Medicine, University Medical Center Ljubljana, Ljubljana, Slovenia; 6 Institute of Health Sciences, Faculty of Medicine, Vilnius University, Vilnius, Lithuania; 7 Centre for Rare Diseases, Faculty of Medicine, University of Debrecen, Debrecen, Hungary; 8 Institute for Medical Genetics and Applied Genomics, University of Tübingen, Tübingen, Germany; 9 Centre for Rare Diseases, University Hospital Tübingen, Tübingen, Germany; 10 Regional Center for Rare Diseases, University Hospital of Udine, Udine, Italy; 11 Centre Léon Berard, University Claude Bernard Lyon 1& Unicancer Lyon, Lyon, France; 12 EURORDIS - Rare Diseases Europe, Paris, France; 13 Medics4RareDiseases, High Wycombe, England, UK; 14 Telethon Kids Institute and the Faculty of Health and Medical Sciences, Division of Paediatrics, He University of Western Australia, Nedlands, WA Australia; 15 Western Australian Register of Developmental Anomalies and Genetic Services of Western Australia, Perth, WA Australia; 16 Rare Care Centre, Child and Adolescent Health Service, Perth, WA Australia

**Keywords:** People living with rare disorders, Rare disease awareness, Medical education and training, Patient empowerment, Interprofessional learning, Highly-specialized knowledge, Social accountability

## Abstract

People living with rare diseases (PLWRD) still face huge unmet needs, in part due to the fact that care systems are not sufficiently aligned with their needs and healthcare workforce (HWF) along their care pathways lacks competencies to efficiently tackle rare disease-specific challenges. Level of rare disease knowledge and awareness among the current and future HWF is insufficient. In recent years, many educational resources on rare diseases have been developed, however, awareness of these resources is still limited and rare disease education is still not sufficiently taken into account by some crucial stakeholders as academia and professional organizations. Therefore, there is a need to fundamentally rethink rare disease education and HWF development across the whole spectrum from students to generalists, specialists and experts, to engage and empower PLWRD, their families and advocates, and to work towards a common coherent and complementary strategy on rare disease education and training in Europe and beyond. Special consideration should be also given to the role of nurse coordinators in care coordination, interprofessional training for integrated multidisciplinary care, patient and family-centered education, opportunities given by digital learning and fostering of social accountability to enforce the focus on socially-vulnerable groups such as PLWRD. The strategy has to be developed and implemented by multiple rare disease education and training providers: universities, medical and nursing schools and their associations, professional organizations, European Reference Networks, patient organizations, other organizations and institutions dedicated to rare diseases and rare cancers, authorities and policy bodies.

## Background

### Unmet needs of people living with rare disorders

In 2010, the Global Independent Commission on the Education of Health Professionals for the 21st Century proposed a systems-based educational reform to improve health system performance and provision of patient and population-centered care [1]. For decades, rare diseases (RD) have been among the priority public health areas in Europe, USA, Japan and some other countries [2–4] and increasingly they are being identified as a global public health priority with growing recognition and calls for action in both high and low and middle-income countries [5–7]. Furthermore, in 2019, the United Nations declared that people living with rare diseases (PLWRD) are among the most vulnerable groups still on the fringes of universal health coverage [8]. In Europe, RD are defined as the ones that affect no more than five persons in 10,000 inhabitants [3], and rare cancers as the ones with an incidence of less than 6 in 100,000 persons per year [9]. Some 6000 to 8000 RD that are currently known may affect any organ or body system at any age, and in most cases RD are multisystem, consequently, physicians of any specialty will see PLWRD. With a point prevalence of 3.5–5.9% (excluding rare infectious diseases, intoxications and rare cancers which represent altogether about 20% of all cancers), PLWRD comprise a significant part of our societies [10], however, still face huge unmet needs. Our health systems are mostly not aligned with the needs of PLWRD and their families [8]; and the healthcare workforce (HWF) lacks competencies to efficiently tackle the challenges and unlock the opportunities for RD [11]. The four main aims of education and training in rare diseases and rare cancers would be to ensure efficient, timely diagnosis and integrated, coordinated, multidisciplinary care, to empower patients and to foster research.

The rarity, diversity, number and heterogeneity of RD create major hurdles to RD recognition in clinical practice. Diagnosis takes 5 to 6 years on average and up to decades [12]. Before achieving a definite diagnosis, PLWRD experience multiple visits to physicians and diagnostic procedures, get misdiagnoses, inappropriate, ineffective or even detrimental treatments [11]. The situation creates intricate diagnostic labyrinths and lengthy diagnostic odysseys, that are tedious, frustrating and costly for patients, their families and health systems. A significant proportion of PLWRD do not get timely diagnosis just due to the lack of awareness and coordinated healthcare systems and pathways [13]: frequently primary or local care professionals do not have a sufficient awareness and index of suspicion for RD, nor the healthcare system literacy for referring patients to the right level and point of care [11, 14, 15].

In addition to the diagnostic challenge, only 5 to 6% of RD have specific, prognosis-changing treatments [12]. This proportion is larger in rare cancers, where close to all cancers have at least proposed treatment options [9, 16]. The fundamentals of the path to the development of effective treatments is the same for both rare and common diseases: there is a need to unveil the mechanisms of a given disease, to identify biological targets for treatments, and to develop drugs or therapeutic procedures. Unfortunately, there is a critical lack of even the basic scientific knowledge for 7 out of every 8 RD, therefore, specific treatments are not even on the horizon for the vast majority of PLWRD, and dedicated research remains scarce for rare cancers [17]. There is a critical need to foster and support RD research, to build the capacities of the entire RD research community and to empower and include patient representatives across the whole research continuum to ensure appropriate tackling of unmet needs.

The multisystem nature of RD demands a multidisciplinary approach, where teams of experts carefully assemble individual signs and symptoms into complex diagnostic puzzles and provide comprehensive and coherent management for PLWRD. About 70% of RD present in childhood and are frequently life-long, disabling and inducing complex needs [18, 19]. Patient and family-centered care is essential in RD with a complex integration of services at multiple levels of health systems and across many medical specialties and sectors, including healthcare, social and educational sectors. Complex trajectories of care pathways and multiple transition points, including life or disease-stage related transitions (e.g., transition from pediatric to adult services or to palliative services) requires meticulous care coordination. Unfortunately, the main burden of care organization and coordination frequently lies on the shoulders of PLWRD and their families and induces significant psychosocial and financial difficulties [20, 21], while the HWF is unprepared to provide a comprehensive, integrated and coordinated, team-work based care for PLWRD and their families [14, 15].

Confronted with the aforementioned gaps, PLWRD and their families become “experts by experience” and acquire multiple roles including that of informal care provider, advocate, case manager, health and social systems’ navigator [22, 23] and even innovator [24]. Although empowered and educated patients better cope with their conditions and have better self-rated health status [25], studies have shed light on the lack of a collaborative relationship between health care providers and PLWRD, where professionals fail to recognize patients and families as informed, involved and equal partners in the care process [26].

In this Statement, we investigate the state-of-the art of knowledge and awareness of RD among the current and future HWF through a comprehensive literature review, identify some of the main challenges for RD education, present a non-exhaustive list of available resources and initiatives in the field, define some general principles of RD education across the continuum of educational stages and specialties and some specific aspects, and call for an action towards a common strategy for RD and rare cancer education in Europe and beyond.

### Level of rare disease knowledge and awareness among the current and future healthcare workforce

Although data are limited, studies to date unequivocally show that future and current HWF lacks even the basic knowledge and awareness of RD (Table 1). In several studies, an objective evaluation of knowledge on RD among physicians (general practitioners and specialists), pharmacists, nurses and students of various specialties was performed by asking questions about RD definition, epidemiology, examples of RD and informational resources for RD [14, 27–36]. Generally, the knowledge on RD was insufficient with correct answer rates for various questions from 2% (question about prevalence of RD) to 91% (question about genetic origin of RD). The vast majority of general practitioners (GPs) and students self-rated poorly their knowledge on RD and preparedness to provide care for RD, while self-ratings of pediatricians and specialists were higher [11, 14, 15, 29, 31, 33–35, 37, 38]. Importantly, in some studies physicians claimed very rare encounters with PLWRD in their practice that may not be compatible with the real RD prevalence rates, hence, it is likely that RD remained unrecognized by responders [27, 37]. In other cases, physicians may not realize their encounters and may underestimate their knowledge of rare diseases or rare cancers: e.g., general practitioners usually have high index of suspicion and relatively good knowledge of “red flags” of pediatric cancer, and all pediatric cancers are rare cancers [39]. According to Australian study, each full time equivalent GP in that country cares for 66 to 86 RD patients in his/her care [40].

**Table 1:** Surveys of current and future healthcare workforce: knowledge on rare diseases

	Objective evaluation of knowledge on RD	Self-rated knowledge of RD	Self-rated readiness to provide care to RD patients	Encountered RD patients in practice	Experienced difficulties in caring RD patients	Educational/ informational sources of knowledge on RD	Awareness where to find information about RD	Awareness where to refer RD patients for specialized services	Awareness about patient organizations	Willingness to broaden knowledge on RD/ expressed need to include mandatory course on RD into university studies	RD seen as a societal and bioethical issue
Walkowiak D, 2020; medical doctors (N 165), PL	Correct answer rates from 15.8% to 53.9%	Insufficient and very poor 94.6%	Rather not and definitely not 93.4%	In practice 75,2%, in family 11,5%		Mandatory courses at university 46.1%, elective courses at university 13.8%, literature 38.9%, conferences 21.6%, Internet 31.7%				Yes 83%/ 76,3%	RD constitute a serious public health issue: 83,1%
Ramalle-Gómara 2020*, GP N 132/ specialists N 37, ES		Likert scale 1 to 5: GP 1.72/ specialists 2.29	Likert scale 1 to 5: qualified to coordinate care GP 1.82/ specialists 2.4		Achieve diagnosis: GP 67,4/% specialists 62,2%; lack of CPG: GP 59,1%/ specialists 70,3%; information about where to refer: GP 66,7%/ specialists 62,2%; lack of access to diagnostic tests: GP 33,3%/ specialists 32,4%	University courses 27%, specialty training: GP 18.9%/ specialists 51.4%; CME courses: GP 40.9%/ specialists 45.9%. Medical training on RD is adequate, Likert scale 1 to 5: GP 1,72/	Likert scale 1 to 5: know patient organizations GP 1.58/ specialists 2.26. specialists 2.29	Likert scale 1 to 5: GP 1.53/ specialists 2.47	Yes: GP 55,6%/ specialists 62,2%. Likert scale 1 to 5: GP 1.58/ specialists 2.26		
Vandeborne 2019, physician, GP N 114/ PED N 95/ specialists 75, BE		Poor and insufficient: GP 86%/ PED 45%/ specialists 16%		At least once: GP 52%. Multiple times: PED 72%/ specialists 94–100%		Usefulness of academic training to diagnose RD; not useful or insufficiently useful GP 80%/ PED 41%/ specialists 7–17%	Yes: GP 27%/ PED 85%/ specialists 75–89% (Orphanet)			Yes GP 84%/ PED 95%/ specialists 95%.; expressed need to include RD courses into university studies: GP 29%/ PED 44%, specialists 39–44%	
Miteva 2011, physicians N 1002, BG	Correct answer rates from 2,3% to 19,8%			During the last year: 4,2%							
Li 2021, physicians N 224, CN; N.B. response rate only 12,4%		Insufficient and poor 94,7%		Overall 53,6%, more than 3 times 19,9%		Education and training for RD sufficient 27,1%				87,8%	Support RD legislation 96,8%
Zurynski 2017, pediatricians 242, AU			Not 28%	Overall 93%, during the last 6 months 74%	Overall 98%; diagnostic delays 65%, lack of available treatments 40%, clinical guidelines 36%, uncertainty where to refer for peer support 35%	University courses 40%, specialty training 50%; consultation with colleagues 92%; Internet 91%; textbooks 49%, mobile phone or tablet applications 30%	Yes 62%	Yes 64%			
Baqué 2019, rare skin diseases GP N 96, FR					Overall: 95%. Achieve diagnosis 88.5%, provide care coordination 76%; lack of knowledge 95%, insufficient time to search for information 72.6%			Know CoE 35.8%			
Mijiritsky 2021, 309 dentists, IL	Correct answer rates 10% to 57.1%			Yes 70,1–95,2%		Medical and specialty training 39.4—77.3%, literature 50.2–69.7%, colleagues 47.6–75.8%					
Kuhne 2020, odontology specialists N 267, DE	Correct answer rates 7.4% to 85.7%, significant differences among university/non-university educated dentists	No or little 50–77,7%		Yes 69–85,7%		No education at university or specialty training 21.4%	Do not know 21.6%, do not need info 10.1%			Yes 98,9%	
Mancuso 2020, neurologists with special interest in RD N 104, IT; N.B. only 4% response rate								Yes 82%; aware of coordinated care in the Region 80%	Yes 73%		Rare neurological diseases are an important disease group: 96%; national health system insufficiently supports rare neurological diseases costs: 25,7%
Krajjnovic 2013, pharmacists N 139, RS	Correct answer rates 33% to 48,2% (2 questions)						Yes 51,8%				Support RD legislation 91,4%; lack of accessibility to Orphan drugs as a problem 64%
Walkowiak 2019, nursing students N 113, nurses 142, PL	Correct answer rates for nursing students 3.5% to 59.3%/ nurses 8.4% to 67.1%	Insufficient and very poor: nursing students 94.7%/ nurses 97.4%	Rather not and not: nursing students 84%/ nurses 77.4%			Mandatory courses at university 10.6%/17.4%, elective courses at university 8%/6.5%, literature 13.3%/21.9%, conferences 6.2%/14.8%, Internet 54.9%/79.4%, I do not search 23.9%/1.3%	Yes (Orphanet) nursing students 0,9%/ nurses 18,1%			Yes nursing students 83.2%/ nurses 91%; expressed need for mandatory educational courses: nurses 85% students 75%	RD constitute a serious public health issue: nursing students 85%/ nurses 92,9%
Ramalle-Gómara 2015, students of various specialties (nursing, medical, non-health) N 234, ES	Correct answer rates from 7.5 to 78.3										Although 72.6% considered that the majority of the budget should be used to treat common diseases, the total mean score for questions about willingness to assign resources to RD ranged from 3.3 to 4.6 on a Likert scale from 1 to 5**
Jonas 2017, students N 270, PL	Correct answer rates 14% to 73.7%										
Domaradzki 2019, students N 346, PL	Correct answer rates 9.5% to 90.5%	Insufficient and very poor 95.4%	No 92.2%			Mandatory courses at university 51.7%, elective courses at university 22%, literature 10.7%, conferences 10.1%, Internet 59.8%, I do not search 11.8%	Yes (Orphanet) 19,4%			Yes 75.1%, but expressed need for mandatory university courses only 54,3%	RD constitute a serious public health problem: 78%, the need for RD legislation: 64,6–74,4%
Domaradzki 2021, students (nursing/ physiotherapy/ medical) N 113/ 173/ 368. N.B. data partially overlaps with Domaradzki 2019	Correct answer rates 3,5% to 89,6%	Insufficient and very poor: 94,7%/ 94,8%/ 95,1%	Rather or definitely not: 84%/ 83,8%/ 91,9%			Mandatory courses at university 10,6%/ 32,4%/ 51,1%, elective courses at university 8%/ 11,6%/ 22,3%, literature 13.3%/ 9,3%/ 19,6%, conferences 6.2%/ 5,8%/ 9,8%, Internet 54.9%/ 53,2%/ 58,7%, I do not search 23.9%/ 17,3%/ 11,4	Yes: 22,1%/ 54,7%/ 39,7%			Yes 83,2%/ 85%/ 73,9% Expressed need for mandatory university course: 76,1%/ 87,9%/ 45,6%	RD constitute a serious public health issue: 85%/ 89%/ 77,2%
Medic 2015, students N 592, Serbia	Correct answer rates 8.2 to 83.05	Likert scale 1 to 10***: 3 to 4				Mandatory courses at university 63.14%, elective courses at university 11.4%, Internet 39.4%					Quality of RD care (Likert scale 1 to 10****): 2,2 to 2,4; importance of RD in society 5,9–5,9

Explanation of Likert scale ratings: *1-poor, 5-very good; **1-not an issue, 5-very important issue; ***1-poor, 10-very good; ***1-bad, 10-very good

The vast majority of physicians experience difficulties in caring for PLWRD, including difficulties to obtain diagnosis, lack of available clinical practice guidelines (CPGs), diagnostic tests, treatments, information about RD and where to refer PLWRD for specialized services, insufficient time to search for information [15, 38, 41]. Awareness of where to find information about RD (e.g., Orphanet) is generally poor among GPs and students and better among pediatricians and specialists. Moreover, a significant proportion of dentists in a study from Germany (10%) and students (up to 24% of nursing students) in a study from Poland claim that they do not need or do not search for information about RD, although general willingness to broaden knowledge on RD is very high [29, 31].

Educational and informational sources of knowledge about RD have also been investigated in a number of studies. About half of all physicians reported university courses and specialty training as an important source of RD knowledge, although usefulness and completeness may be limited (academic training not useful or insufficient for 7–17% of specialists and 80% of GPs) [11]. Nursing and physiotherapy students may receive even lower university education on RD when compared to medical students [34]. Interestingly, despite a general willingness to broaden knowledge on RD diseases and claims about insufficient academic RD education, respondents were relatively reluctant for inclusion of mandatory RD university courses (expressed need for such course: 29% of GPs, 44% of pediatricians, 39% to 44% of specialists, 85% of nurses, 46% to 88% of students [11, 31, 33]); this may be due to a combination of both lack of awareness of the magnitude of burden of RD [42] and the perception of already overwhelmed curricula. As expected, continuous medical education, scientific literature and conferences are considerably more important as a source of information on RD for practicing physicians and nurses; many of them completed their studies a number of years ago, when RD concepts where not sufficiently developed [31, 34]. However, perhaps unexpectedly, the Internet was mentioned as an important source of information about RD by a considerably higher number of practicing professionals as compared to students; presumably, professionals are forced to search for information about RD when they encounter suspected or confirmed RD cases in their practice [31, 34, 38].

Several studies of knowledge base in certain RD groups presented similar results [28, 29, 43–46] (Table 1). A study performed by the European Reference Network on rare endocrine diseases, Endo-ERN, investigated knowledge base on rare endocrine diseases [47]. The largest knowledge gaps were reported for GPs (71%), followed by students and medical specialist trainees (61% each), and specialists (51%). Out of 146 respondents across 19 European Union (EU) Member States, only 45% had a structured RD educational plan, and only 36% reported a specific training program for GPs. There was an almost unanimous desire for a more harmonized approach towards education and training through the common e-learning platform of professional organizations (European Society of Pediatric Endocrinology and European Society of Endocrinology) and the Endo-ERN.

Several studies investigated knowledge base in rare cancers. In a study performed by the Joint Action Rare Cancers and the European Union of Medical Specialties (UEMS), 104 respondents of all European nations were questioned about education and training in rare cancers, including undergraduate and postgraduate training [48]. Only a small proportion of respondents had received specially dedicated undergraduate teaching (19%) or targeted teaching materials (26%) for rare cancers. Knowledge and awareness on rare cancers of a training personnel in the institution or country was frequently rated as poor (accordingly, in 20% and 30% of cases). Similarly, ratings of knowledge and awareness of rare cancers among the new MD graduates were generally low (poor knowledge: 43% of respondents). Additionally, more than half of the participants did not feel that GPs are aware and well informed on rare cancers (56%), while ratings of pediatricians (not aware 21%) and specialists (11%) were more favorable. There was a general agreement that European training in rare cancers is fragmented (77% of respondents agree) and there is a need for pan-European harmonization of training (90% of respondents agree).

### Rare disease education challenges

Although the need for RD education for the current and future HWF is evident from both public health (unmet needs of PLWRD and families) and learners’ perspective (objective and self-reported insufficiency of RD knowledge), there is a general lack of attention to developing and delivering targeted and coordinated RD education that may be attributed to multiple factors. For many decades RD were, and in many cases still are, neglected in healthcare systems; due to the lack of specific RD codification, the RD burden is systematically underestimated contributing to the relative invisibility of its public health impact [49], although for rare cancers better codification opportunities and consequently more data on burden are available [50]. Together with a paradox of rarity (i.e., RD and rare cancers are individually rare, but collectively common), it may create a perceived lack of interest towards RD; indeed, both professionals and students usually severely underestimate probabilities of encountering RD in their practice [51]. A frequent complaint of both educators and learners are overwhelmed curricula and lack of time in the extremely busy agenda of practicing clinicians and nurses; although the digital transformation has changed the role of universities from memorization of facts to location of requisite information for synthesis, analysis and decision-making, the sheer amount and dispersion of novel data and information in medicine are immense and RD field is no exception with 6000 to 8000 nosological entities currently known. Having in mind the sheer number of RD, lack of time and already overwhelmed academic curricula, it seems very appealing to prioritize RD education based on, e.g., prevalence (149 RD comprise 77.3–80.7% of the population burden of RD [10]) or treatability [52, 53]. Although for some groups of stakeholders, e.g., general practitioners or medical students, it could be of relevance, caution should be taken not to leave any RD behind as it could increase inequity among PLWRD. An important challenge in medical education on RD and rare cancers, that makes it essentially different from common diseases, is the lack of reinforcement of information for the vast majority of HWF: after gaining some knowledge on a certain RD or rare cancer, practitioners do not encounter this disease in their practice for many years or not at all. Therefore, complementary educational strategies and goals should be applied to professionals across the education and training continuum from generalist to specialist and expert. Moreover, RD as a field has novel dynamics; in Europe, RD have been defined and prioritized as a public health issue only two decades ago and many current professionals and educators have completed their medical education long before. The huge heterogeneity and multisystem nature of RD present additional challenges: although the gaps and needs for various specialties and types of professionals (eg, physicians and nurses) are largely unexplored, RD education is important to everybody in care systems. Moreover, with improved treatments, many PLWRD that previously had severely limited survival are living well into adulthood nowadays; there is a crucial lack of knowledge on many aspects associated with prolonged survival and novel treatments, including late adverse effects of interventions, definition of new natural histories or effects of aging on certain RD [54]. Unfortunately, there is a lack of investment in RD not only among academic institutions, but also among many professional organizations that play an important role in definition of educational standards and provision of continuous medical education, although some of them have developed educational resources for RD and rare cancers recently in collaboration with ERNs (Table 2) [55]. Some RD education is provided by a private sector, in many cases this education targets just one RD or a small group of all RD and may create some issues of RD education independent governance and increasing inequities among PLWRD. Finally, although many on-line, face-to-face or blended resources for RD education and training have been developed in recent years (Table 2), awareness about these resources is still very low [56].

**Table 2:** Non-exhaustive list of on-line rare disease knowledge, education and training resources

Type of resources	Knowledge, education and training resources	Description
Knowledge bases, the main organizations for rare diseases	Orphanet; https://www.orpha.net/consor/cgi-bin/index.php	Portal for rare diseases and orphan drugs
	OMIM^a^; https://www.omim.org/	Online Catalog of Human Genes and Genetic Disorders
	EURORDIS; https://www.eurordis.org/	Alliance of patient organizations representing > 900 rare disease patient organizations in >70 countries
	NORD; https://rarediseases.org/	Umbrella of > 300 patient organizations
	European Medicines Agency. Orphan designation: overview; https://www.ema.europa.eu/en/human-regulatory/overview/orphan-designation-overview	Information about Orphan medicines in the EU
	IRDiRC; https://irdirc.org/	International Rare Diseases Research Consortium that promotes international collaboration and advance RD research worldwide
	GeneReviews; https://www.ncbi.nlm.nih.gov/books/NBK1116/	Information on inherited conditions in a standardized journal-style format, covering diagnosis, management, and genetic counseling
European Reference Networks	Information and links: https://www.orpha.net/consor/cgi-bin/Clinics_ERN.php?lng=EN; https://ec.europa.eu/health/ern_en ERN on rare bone diseases: ERN BOND; https://ernbond.eu/ ERN for rare and/or complex craniofacial anomalies and ear, nose and throat (ENT) disorders: ERN CRANIO; https://ern-cranio.eu/ ERN on rare endocrine disorders: Endo-ERN; https://endo-ern.eu/ ERN on rare and complex epilepsies: EpiCARE; https://epi-care.eu/ ERN on rare kidney diseases: ERKNet; https://www.erknet.org/ ERN on rare neurological diseases: ERN-RND; https://www.ern-rnd.eu/ ERN on rare and congenital anomalies: ERNICA; https://ern-ernica.eu/ ERN on rare respiratory diseases: ERN-LUNG; https://ern-lung.eu/ ERN on rare and undiagnosed skin disorders: ERN-Skin; https://ern-skin.eu/ ERN on rare adult solid tumours: EURACAN; https://euracan.eu/ ERN on rare hematological disorders: EuroBloodNet; http://www.eurobloodnet.eu/index/ ERN on rare neuromuscular diseases: EURO-NMD; https://ern-euro-nmd.eu/ ERN on rare eye diseases: ERN-EYE; https://www.ern-eye.eu/ ERN on genetic tumour risk syndromes: GENTURIS; https://www.genturis.eu/l=eng/Home.html ERN on rare urogenital disorders: eUROGEN; https://eurogen-ern.eu/ ERN on rare and complex diseases of the heart: ERN GUARD-Heart; https://guardheart.ern-net.eu/ ERN on rare congenital malformations and rare intellectual disability: ERN ITHACA; https://ern-ithaca.eu/ ERN on inherited metabolic diseases: MetabERN; https://metab.ern-net.eu/ ERN on pediatric cancer: ERN PaedCan; https://paedcan.ern-net.eu/ ERN on rare hepatological diseases: ERN RARE-LIVER; https://rare-liver.eu/ ERN on rare connective tissue and musculosceletal disorders: ERN ReCONNET; https://reconnet.ern-net.eu/ ERN on rare immunodeficiency, autoinflammatory and autoimmune diseases: ERN RITA; https://ern-rita.org/ ERN on transplantation in children: ERN TransplantChild; https://www.transplantchild.eu/ ERN on rare multisystemic vascular diseases: VASCERN; https://vascern.eu/	
Professional organizations^b^	European Union of Medical Specialties (UEMS): Multidisciplinary Committee on Rare and Undiagnosed Diseases (MJC RUD); https://uems-genetics.org/links.html	Develops competency training requirements and syllabuses for rare and undiagnosed diseases and rare cancers, organizes European examinations
	Society for the Study of Inborn Errors of Metabolism (SSIEM); https://www.ssiem.org/training	Organize SSIEM Academy courses on inherited metabolic diseases
	Rare Cancers Europe; https://www.rarecancerseurope.org/events	Training courses for patient advocates in rare cancers, developed together with ESMO and ESO
	European Society of Human Genetics (ESHG)^a^; https://www.eshg.org/index.php?id=education	Provides courses and various educational resources for human genetics
	International Society of Pediatric Oncology (SIOP); https://casehippo.com/spa/courses/catalog/siop/home	SIOP Knowledge Centre provides educational resources on pediatric cancers
	International Society of Amyloidosis; https://www.isaamyloidosis.org/meetings-education	Provides workshops and seminars on amyloidosis
	European Society for Pediatric Nephrology (ESPN)^a^; https://www.espn-online.org/espn-ipna-erknet-educational-best-clinical-practice-webinars/#	Provides webinars and other educational resources on rare pediatric kidney diseases (in collaboration with ERN ERKNet)
	European Society of Endocrinology^a^ https://www.ese-hormones.org/about-us/committees/rare-disease-committee/ and European Society of Pediatric Endocrinology^a^ https://www.eurospe.org/education/	Provides some education and awareness raising on rare endocrine diseases (in collaboration with Endo-ERN)
	International League Against Epilepsies^a^; https://www.ilae.org/education	Provides some e-learning modules on rare epilepsies
	European Academy of Neurology^a^; https://www.ean.org/learn/joint-webinars	Provides educational programme (developed in collaboration with ERN-RND and ERN-EuroNMD)
	European Respiratory Society^a^; https://www.ers-education.org/collections/educational-material-on-rare-diseases/	Provides some educational e-learning materials for rare respiratory diseases (developed in collaboration with ERN-LUNG)
	European Society of Medical Oncology^a^ (ESMO)	Provides some educational resources on rare cancers (in collaboration with EURACAN)
	European Hematology association; https://ehaweb.org/education/	Provides some educational resources on rare hematological diseases (in collaboration with EuroBloodNet)
	European Association for the Study of the Liver^a^; https://easlcampus.eu/ern-on-demand	Provides webinars on rare liver diseases (in collaboration with ERN RARE-LIVER)
Education and training resources^b^	ESHG Genetic Educational Materials and Sources^a^; https://www.eurogems.org/index.html	A compendium of genetic information and resources
	European School of Oncology^a^; https://www.eso.net/	Organization for education and training in cancer
	EURORDIS Open Academy; https://openacademy.eurordis.org/	Capacity-building programmes for patient advocates and mixed audiences
	BBMRI.QM Academy^a^; https://www.bbmri-eric.eu/services/e-learning/	E-learning resources on biobanking
	Elixir Training Platform^a^; https://elixir-europe.org/platforms/training	Education and training resources on life sciences
	EATRIS Transmed Academy—course on translational medicine^a^; https://eatris.eu/services/education/^a^	e-learning platform which hosts online courses as well as recordings of webinar series
	European Patients’ Academy Webinars^a^; https://www.eupati.eu/category/webinar/	12 webinars from EUPATI, for patients and advocates
	European Patients’ Academy Expert Training Course^a^; https://www.eupati.eu/eupati-training-course/	Online course in medicines research and development. Not solely focused on rare diseases
	Integrated DEsign and AnaLysis of clinical trials in small population group (IDeAl) resources; https://www.ideal.rwth-aachen.de/?page_id=1732	Webinar Series on Integrated DEsign and AnaLysis of small population group trials
	Research Data Management online courses^a^; https://vidensportal.deic.dk/en/RDMELearn	eLearning course about the importance of good research data management (RDM)
	Patient-Centered Outcomes Research Institute (PCORI) Training: A Program for Rare Disease Patient Advocates; https://www.pcori.org/research-results/2015/pcor-training-program-rare-disease-patient-advocates	Tools and templates on the subject of "patient and research"
	Findacure's e-learning resources on rare diseases; https://portal.findacure.org.uk/	The portal is aimed at rare disease advocates, patient groups and charities, it shares ‘how to’ and best practice on a range of topics-from building the team to running patient registries-to encourage efficient and sustainable growth of patient groups
	FutureLearn courses on genomics^a^: The Genomics Era: the Future of Genetics in Medicine https://www.futurelearn.com/courses/the-genomics-era; Whole Genome Sequencing: Decoding the Language of Life and Health; https://www.futurelearn.com/courses/whole-genome-sequencing; Genomic Technologies in Clinical Diagnostics: Next Generation Sequencing; https://www.futurelearn.com/courses/next-generation-sequencing; Genomic Technologies in Clinical Diagnostics: Molecular Techniques; https://www.futurelearn.com/courses/molecular-techniques	Courses on genomic technologies, whole genome sequencing
	Genetics education for primary care resources from the Gen-Equip project^a^ https://www.primarycaregenetics.org/?page_id=109&lang=en	Genetics education for continuing medical or professional education in genetics (general practitioners, primary care pediatricians, midwives, and primary care nurses)
	Medics4RareDiseases (M4RD) video library; https://www.m4rd.org/video-library/	e-learning for medical students and doctors about the fundamentals of rare diseases
	Program on rare diseases “Excellence In pediatrics”; https://www.ineip.org/p2p_education_program_on_rare_diseases_excellence_in_pediatrics	Peer-to-Peer Education Program on Rare Diseases including Live Lectures, Enduring Online Content & Community-Based Education
	Recordati rare diseases; https://www.rrd-foundation.org/en/courses	Courses and webinars on clinical trials and rare diseases (mostly inherited metabolic diseases)
	Aarhus University, Rare Diseases in Translational and Personalized Medicine; https://kursuskatalog.au.dk/en/course/105020/Rare-Diseases-in-Translational-and-Personalized-Medicine	MsC course on rare diseases, translational and personalized medicine
	Wellcome Advanced Courses and Scientific Conferences-Genomics of Rare Disease; https://genetics.org.uk/events/wellcome-advanced-courses-and-scientific-conferences-genomics-of-rare-disease/	Courses on rare diseases, genomics, undiagnosed diseases
	Queen’s University Belfast; https://www.qub.ac.uk/sites/RareDisease/Events/	Courses, seminars and webinars on rare diseases

^a^Resources that are not specific for rare diseases but include important aspects on rare diseases

^b^Some of these resources may have limited duration or accessibility

### State-of-the-art in rare disease education: available resources and the needs across education and training continuum

**Fig. 1:**
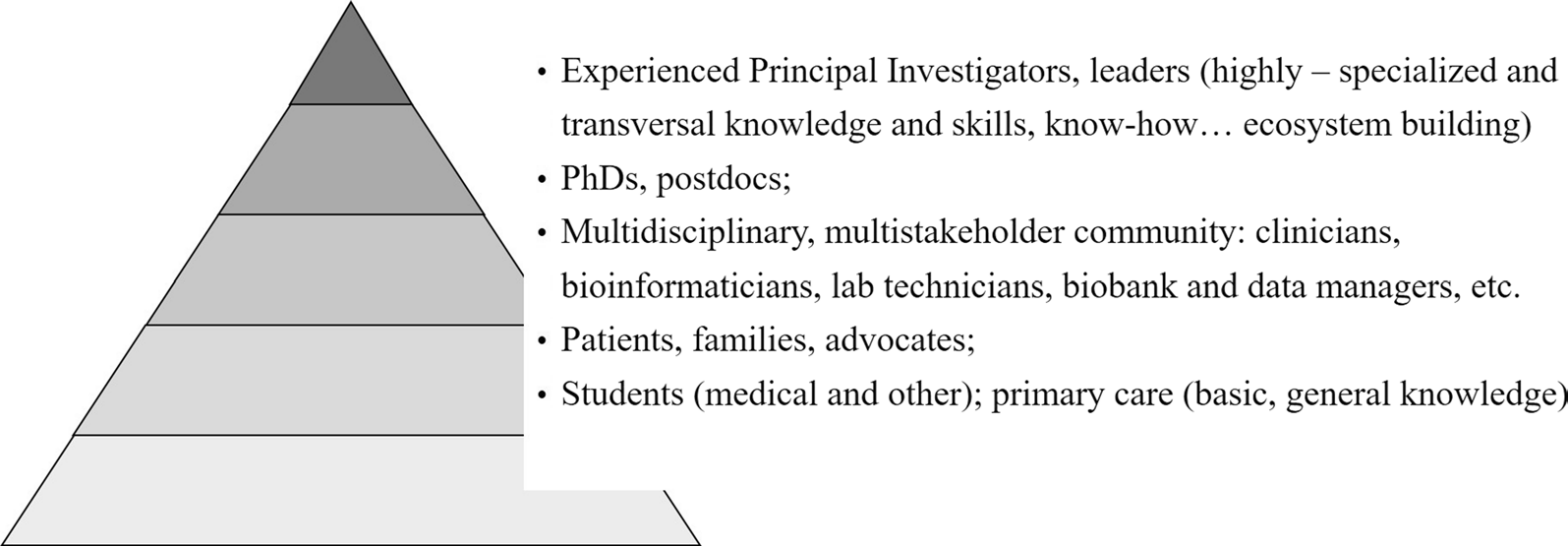
Principle of pyramid in rare disease education and training. The basis of the pyramid comprises a general knowledge base and includes vast groups of stakeholders as students and general practitioners; the top of the pyramid refers to highly-specialized knowledge and knowledge generation and includes experts and thought leaders

There is no health without the healthcare workforce: if we want to respond to unmet needs of PLWRD and their families, we need to fundamentally rethink RD education and HWF development. The entire spectrum that makes up the HWF requires knowledge and awareness of RD, but the needs are vastly different across the education and training continuum and follows a principle of a pyramid (Fig. 1). The vast majority of the HWF, including students, nurses and GPs, is at the basis of this pyramid; they have to be equipped with the basic, general knowledge about RD (like „red flags “ to recognize RD, the most common RD, awareness on where to refer a patient for specialized services, skills for participation in long-term management, integrated care and care coordination). Highly-specialized experts are situated at the very top of the pyramid; their education and training takes much more time and efforts, includes not only formal, but also informal and non-formal training [57], and they are not only “learners”, but also generators of expertise and knowledge. Middle layers of the pyramid involve the whole multistakeholder community with highly variable needs according to the specialty and scopes of practice, like specialists, nurse coordinators, multidisciplinary team members, researchers. Importantly, PLWRD and their advocacy organizations should be included at all stages of the pyramid: they need knowledge and skills for the empowerment, active and meaningful participation in self-care, and advocacy and leadership skills to engage and partner with HWF, researchers, policy makers and regulatory agencies. As PLWRD are experts of their own disease, they could be consulted and engaged into the creation and alignment of educational contents to unmet needs, provision of medical and peer-to-peer education [58–60].

This education and training continuum involves multiple forms of teaching and learning, including formal, informal and non-formal education, vocational training and development of skills through lectures, seminars, bed side teaching, case reports, case scenario discussions, journal clubs, e-learning, webinars, fellowships, computer assisted, self-instruction modules, problem-based learning, team-based learning, simulation, etc. Universities, nursing and medical schools should be the main providers of basic and general knowledge on RD for the future HWF, while teaching hospitals implement clinical and specialty education and training. As a large part of current HWF lacks RD awareness and knowledge, continuous medical education is required to fill this gap. Professional organizations define educational standards for their specialty, provide continuous medical education and other resources (e.g., on-line training modules, conferences and educational events) for professional development, and have a special role in filling the knowledge gaps of current HWF (Table 2). Recently, a Multidisciplinary Joint Committee on Rare and Undiagnosed Diseases was established in UEMS and developed competency training requirements and syllabuses for rare and undiagnosed diseases [61] and rare adult solid cancers [62]. These resources may aid in the harmonization of training requirements across Europe and beyond and may serve as a base for RD and rare cancers education in the national educational systems.

Highly-specialized knowledge and skills of RD experts are often acquired in the Centers of Expertise (CoE) or Comprehensive Cancer Centers [63]. In 2017, more than 900 of CoE joined their forces and created 24 European Reference Networks (ERNs) across the main RD and rare cancers domains. Currently ERNs include more than 1600 CoE and, through EU-wide leveraging of existing educational and training resources, generation of knowledge and development of novel educational and training means, provide powerful resources for highly-specialized RD knowledge and expertise (Table 2) [64–66]. Additionally, through the involvement of patient organizations and European patient advocacy groups (ePAGs), ERNs play an important role in the development and provision of educational resources for patient empowerment [58, 60, 67]. ERNs organize their educational strategy via the ERN Knowledge Generation working group formed by ERN coordinators and representatives of the ERN Board of Member States [68], aimed at the development of common approaches to promote and sustain courses, masterclasses, post-doctoral programs and mobility programs for pre- and postdoctoral fellows on RDs. All these activities constitute the ERN Academy, a virtual platform which will collect all the educational modules generated by the ERNs.

Education on RD research is provided by research institutions and research infrastructures, like European Research Infrastructure for biobanking BBMRI-ERIC [69] or European Research Infrastructure for life sciences ELIXIR [70]. A recently developed European Joint Programme on Rare Diseases (EJP RD) encompasses a comprehensive RD research education and training programme that aims to fulfill the educational needs of the RD research multistakeholder ecosystem along the whole research and innovation pipeline (i.e., basic to preclinical to clinical research and translation) and across career stages (from students to principal investigators) (Table 2) [71]. Patient organizations play an indispensable role in the education, empowerment and capacity building of PLWRD, families and advocates. Through it ‘s extensive patients ‘ and mixed audiences-targeted (including both patient representatives and other multistakeholder community members as clinicians and researchers) training programmes, the largest alliance of currently 974 RD patient organizations EURORDIS equips trainees with crucial leadership, advocacy and partnership-building skills for a meaningful inclusion of RD patient representatives into all RD-related activities [71]. Finally, important educational resources are also provided by some non-governmental organizations as Medics4RareDiseases that leverages on the social accountability and involvement of medical students and young professionals to foster RD education [72].

### Some specific aspects of rare disease education and training

#### Care coordination: the role of case managers and nurse coordinators

Care coordination for PLWRD is highly complex: there is a need to ensure better navigation through complex trajectories and smooth transitions across health, social care, educational systems, among many professionals, and across the lifespan and disease stages. PLWRD and caregiver experiences of care are vastly negative [18, 20–23, 73–75]. Although some aspects of integrated, coordinated care for PLWRD and families have been defined [18, 21, 76, 77], implementation of care coordination depends on dedicated, educated and empowered professionals-case managers or nurse coordinators. Nurse coordinators successfully implement a variety of care functions, including care coordination, holistic oversight, symptom and adverse event monitoring and management, and emotional support, in care of patients with cancer or chronic diseases [78, 79], however, their role in RD care coordination is still underinvestigated and there is a lack of dedicated educational programmes.

#### Interprofessional training for integrated multidisciplinary care

Although the concept of interprofessional education (IPE) as a means to decrease health systems ‘ fragmentation and ensure integrated care was proposed a number of years ago [80], it ‘s implementation is often hampered by „uniprofessional identity “, persistent negative stereotypical attitudes towards other professionals and practical issues such as integration of IPE into curricula of different educational programmes, alignment of scheduling and logistics, staffing and funding for IPE [81–83]. However, due to the need for complex integrated care and multisystemic nature, RD could be an excellent case study for interprofessional training that equips HWF with crucial skills and capabilities. A salient example of an increased partnership between health and educational sectors is a recently launched mEDUrare initiative that has begun aligning policy, practice and workforce training between the Health and Education systems in Western Australia [84].

#### Patient- and family-centered rare disease education: „expert patients “and patient empowerment

PLWRD and caregivers are frequently exceptionally knowledgeable: a proportion of PLWRD thus often know more about their disease than the professionals whom they meet on their care journey [58]. These role discrepancies may have a negative impact on patient-physician relationship and communication processes [26, 85]. Hence, the success of the relationship crucially depends on the professional ‘s ability to acknowledge the active role of the patient as an informed, involved and equal partner in the care process and gain mutual trust through the sensitive, empathetic, transparent, supportive for proactivity, human communication and attitudes [26, 86]. There is a need to equip future HWF with appropriate communication skills, professional values and attitudes for the improvement of PLWRD and caregivers’ experiences [76].

PLWRD and caregivers have high needs for information and skills in self-management, coping, communication and advocacy [60]. Hence, there is a need to develop patient empowerment and educational programmes that may be provided by specialist nurses, allied health or other highly-specialized professionals [60, 67]. Collaboration with patient organizations may also play a highly important role in the fulfillment of patients ‘informational and educational needs and the establishment of partnerships among patients and professionals, while one of the most effective ways to ensure partnerships may be through the integrated learning of mixed audiences (patient representatives and professionals together). EURORDIS Open Academy trained over 600 RD patients and patient advocates since 2008, empowering them with knowledge and skills to take part in patient engagement roles side-by-side with all stakeholders and to advocate for rare diseases on a European and national level [71, 87]. Besides, as the experts of their own disease, PLWRD may provide valuable information on unmet needs and fill the gaps of missing information that is available to various professionals on their care journey [58].

#### The power of e-learning and the need for digital literacy

Digital transformation affects both healthcare and educational sectors. In RD care, it is critical to overcome geographical barriers and provide remote, even cross-border health services [88, 89], empower PLWRD [71], and interconnect RD expertise and knowledge [68, 90]. Digital health solutions are indispensable for improved RD diagnosis, treatment, navigation and care coordination, and integration and coordination for broader societal and patient wellbeing [91]. During recent years, many valuable on-line RD educational and informational resources have been developed by ERNs, EJPRD, EURORDIS, European Society of Human Genetics, European School of Oncology, Medics4RareDiseases and other stakeholders (Table 2). The importance of these resources cannot be underestimated: high quality on-line information for both professionals and PLWRD is a high unmet need [92], besides, it may counterbalance low quality or even detrimental potential of some Internet and social media sources [93, 94]. While the recent COVID-19 pandemic has induced major disruptions in both healthcare and educational systems, advances in digital technologies provided crucial means to overcome at least some of the challenges and may provide an enduring basis for long-term changes [95, 96], and RD are an exemplar domain for advancing and implementing digital (health) technology [91]. Notably, the major drivers for a constructive digital transformation are not only the hardware or software, but the so-called humanware [97]; hence, there is a high need to equip HWF with the crucial digital skills and to evaluate further potential of digital RD education and training under both normal and emergency conditions.

#### Professionalism, social accountability and culturally safety and responsiveness

The recent movement towards socially accountable professionalism [1] and value-based care [98] enforces the focus on socially-vulnerable groups such as PLWRD and calls for strengthening of transversal “soft” skills to equip professionals with analytical, leadership and communication capabilities and instill a culture of life-long learning. Both current and future HWF generally acknowledge RD as a significant public health issue that requires some special measures (Table 2) [14, 15, 30, 31, 33, 35, 37, 43, 99]. Education is required to support equitable health care delivery through culturally safe and responsive care. This includes, but is not limited to, how best to support, respect and work together with: different languages, customs and narratives; address other access barriers including stigmatization and referral bias; understanding of the challenges of limited knowledge of (gen)omic and phenotypic reference data and the importance of indigenous data sovereignty and related principles and enable capacity building and empowerment of Indigenous people, community and workforce [100].

## Conclusions

### Next steps: towards a common strategy for rare disease and rare cancer education and training in Europe and beyond

In order to fill the knowledge and awareness gaps of current and future HWF and to equip HWF and multistakeholder community of RD and rare cancers with strong knowledge base and skills to deal with manifold RD and rare cancers ‘ challenges, there is a need for a common strategy on education and training in Europe and beyond. It has to be developed and implemented by multiple education and training providers: universities, medical and nursing schools and their associations, professional organizations, European Reference Networks, patient organizations, other organizations and institutions dedicated to RD and rare cancers, authorities and policy bodies (Table 2). All of them together provide coherence and complementarity for the education and training across the whole RD and rare cancers ecosystem. International collaboration is indispensable in RD and rare cancers and provides the means for harmonization of educational standards across different countries and globally. There is a need to identify gaps and needs across many domains: for various professions and specialties, and according to the learners’ perspective based on the principle of pyramid (Fig. 1). As the areas of RD and rare cancers share many commonalities, but also some important differences, there is an opportunity for sharing some best practices and solutions between the two areas and the need to identify sometimes different strategies for education and training (e.g., diagnostic urgency is much more important in rare cancers and requires teaching on “red flags”). For every group of stakeholders and diseases, educational outcomes have to be clearly defined and educational programmes and frameworks developed (including training requirements, syllabuses, educational resources, etc.). Finally, national, international and professional policies and strategies have to support RD and rare cancers education and training, like the recently developed ERN Strategy on education and training and prioritization of rare diseases as one of the strategic directions in the Association of Medical Schools in Europe.

## Data Availability

Please contact author for data requests.

## Abbreviations

CoE: Center of expertise
CPG: Clinical practice guidelines
EJP RD: European joint programme on rare diseases
ePAGs: European patient advocacy groups
ERN: European reference network
ESHG: European society of human genetics
EU: European union
GP: General practitioner
HWF: Healthcare workforce
IPE: Interprofessional education
MD: Medical doctor
PLWRD: People living with rare diseases
RD: Rare diseases
UEMS: European union of medical specialties
USA: United States of America
